# Discriminating between Absorption and Scattering Effects in Complex Turbid Media by Coupling Polarized Light Spectroscopy with the Mueller Matrix Concept

**DOI:** 10.3390/s22239355

**Published:** 2022-12-01

**Authors:** Arnaud Ducanchez, Maxime Ryckewaert, Daphne Heran, Ryad Bendoula

**Affiliations:** 1 ITAP, University of Montpellier, INRAE, Institut Agro, 34060 Montpellier, France; 2 ITAP, University of Montpellier, INRAE, 34196 Montpellier, France

**Keywords:** spectroscopy, polarization, Mueller matrix, complex media, absorption, scattering, PLS

## Abstract

The separation of the combined effects of absorption and scattering in complex media is a major issue for better characterization and prediction of media properties. In this study, an approach coupling polarized light spectroscopy and the Mueller matrix concept were evaluated to address this issue. A set of 50 turbid liquid optical phantoms with different levels of scattering and absorption properties were made and measured at various orientations of polarizers and analyzers to obtain the 16 elements of the complete Mueller matrix in the VIS–NIR region. Partial least square (PLS) was performed to build calibration models from diffuse reflectance spectra in order to evaluate the potential of polarization spectroscopy through the elements of the Mueller matrix to predict physical and chemical parameters and hence, to discriminate scattering and absorption effects, respectively. In particular, it was demonstrated that absorption and scattering effects can be distinguished in the Rayleigh regime with linear and circular polarization from the M_22_ and M_44_ elements of the Mueller matrix, correspondingly.

## 1. Introduction

Visible and near-infrared (VIS–NIR) spectroscopy is an approved analysis technique for measuring the chemical composition of a very wide variety of media and products. It is a way of obtaining information in a fast, accurate and non-destructive manner. Nowadays, this powerful method is commonly used in routine analysis, in-line in industries or in-field, and has found considerable applications in biomedical [1], agricultural [2,3] and environmental [4] domains.

By examining spectrum attenuation through the absorptive media, it is possible to extract chemical information such as the concentration of the absorbing substance using the Beer–Lambert law, which has a linear relationship between spectral absorbance and low concentrations of chemical species. However, this ideal case is subject to several assumptions. One critical prerequisite is that the absorption pathlength must be well-defined, which means that the scattering must be negligible. Hence, the Beer–Lambert law is not applicable to complex media where the light is not only absorbed but also substantially scattered.

Unfortunately, in practice and whatever the application fields, the majority of the media studied and analyzed are so-called “turbid media”, where light absorption and scattering effects are mostly observed together in these complex media. As a result, the spectra are often impacted by numerous phenomena other than chemical components of interest caused mainly by scattering. This may result in non-linearity with multiplicative and additive effects in the spectral data due to physical effects that disturb and mask the spectral variations related to the chemical parameters that can change between different samples. These physical effects, resulting from the variation in optical path length, are due to physical differences between samples such as particle size and shape, sample surface and packing [5]. Therefore, to overcome the problem of multiple scattering and non-linearity in turbid and highly diffusive media, different strategies have been proposed in the literature to reduce, eliminate or separate the scattering and absorption effects in VIS–NIR spectra.

The most common approach is based on advanced chemometric methods to minimize scattering effects through the use of spectral pretreatments [6,7]. These pretreatments are specifically dedicated to correcting additive and multiplicative effects on the spectra due to uncontrolled variations of physical properties in the measured samples. One of the most widely used preprocessing techniques on VIS–NIR spectra is grouped under the name of “scatter-correction methods” [7]. This category of scatter-corrective preprocessing methods includes standard normal variate (SNV) [8], multiple signal correction (MSC) [9] and normalization [10]. These different methods are often associated with baseline correction methods such as detrend, derivatives (Savitzky–Golay, Norris–Williams, etc.) or asymmetric least squares (AsLs). Although effective, these different approaches remain debatable as they are based on underlying assumptions such as the fact that scattering is almost constant over the entire wavelength range, which is not the case. Furthermore, they are inappropriate when light scattering varies considerably from sample to sample. Lastly, although preprocessing can be very useful, there is always a trade-off between information loss and noise reduction. Indeed, by eliminating scattering effects, the chemical signal can also be attenuated or even altered.

Therefore, another more rigorous approach that uses the fundamentals of light propagation through radiative transfer theory has been investigated over the last years [11,12]. This approach involves the use of the radiative transfer equation (RTE) to decorrelate scattering and absorption effects through specific experimental techniques including double integrating sphere setup [13,14], and spatially resolved [15], time-resolved (also called photon time-of-flight method) [16] or frequency-resolved spectroscopy [17]. Although the potential is evident, there are some limitations and challenges to overcome when these different techniques based on the RTE method are applied on highly turbid samples [18]. Moreover, all these techniques are not appropriate for easy incorporation into conventional multi-spectral or spectrometer devices due to their cost and/or complexity.

At last, another recent approach based on speckle measurement has shown promising results, which relies in part on the use of polarized light [19]. However, this proposed method has also shown some limitations in the prediction models of optical absorption and scattering coefficients (µ_a_ and µ_s_) according to the media and wavelength considered.

For a long time now, polarized light interaction with turbid media due has received considerable interest due to its possibility to reduce multi-scattering effects of the measured signal [20], especially in biomedical applications [21].

In fact, a simple technique based on polarization subtraction can be used to extract the light that preserved its initial polarization state, which contains useful information even if present in small quantities [22]. However, the majority of studies are based on the use of one polarization state (linear, circular or elliptical) [23], without any combination of these different states of polarization. On the other hand, the Mueller matrix is well-known to describe complete information about all the polarization properties of media and materials [24]. Some studies using this concept are focused on the particular elements of the Mueller matrix, or else, on all the elements of the Mueller matrix but with a single wavelength or only based on polarization imaging [25,26].

This work follows a first article on valorization of the spectral database resulting from experimentation [27]. In this paper, the main objective was to exploit this database and to explore the potential of VIS–NIR spectroscopy in polarized light combined with the Mueller matrix concept with multivariate analysis based on PLS methods in order to predict absorption and scattering properties through the evaluation of matrix elements. In particular, this study focused on the diagonal elements characteristic of depolarization properties to discriminate absorption and scattering effects through the establishment of predictive models from absorber and scatterer concentrations of model turbid media, respectively. One of the final objectives in implementing this method was to decrease detection limits to ultratrace levels. In fact, within some applications in the fields of environment, agri-food and agriculture, the problems of the detection and quantification of various contaminants and pollutants at very low concentrations still remains challenging in some case. Thus, this original optical approach could therefore be applied to address these issues.

## 2. The Mueller Matrix

This concept, known as the formalism of Mueller matrices or as the Stokes–Mueller formalism, was introduced by H. Mueller in 1943 [28]. The Mueller matrix M gives a complete description of how an optical media interacts and/or transforms the polarization state of an incident light beam in either reflection or transmission configurations. This 4 × 4 real matrix completely characterizes any product or media in terms of its optical properties through the interaction of polarized light with matter and can be considered as the “optical fingerprint” or transfer function of a media.

The Stokes parameters describing the polarization state are often combined into a vector, known as the Stokes vector. In practice, the polarization state of the light leaving the medium is the result of the transformation of the Stokes vector between the input and output of the medium. This transformation is represented by the Mueller matrix, which is defined by the following linear relationship:

*S*_0_ = *M* · *S*_*i*_ (1)

where *S*_0_, *S_i_* and *M* are the output Stokes vector, the input Stokes vectors and the Mueller matrix, respectively. The Stokes vector (*S*) and the Mueller matrix (*M*) are defined in Equations (2) and (3), respectively:

(2) $$S=\left[\begin{array}{c} I\\ Q\\ \begin{array}{c} U\\ V \end{array} \end{array}\right]=\left[\begin{array}{c} I_{H}+I_{V}\\ I_{H}-I_{V}\\ \begin{array}{c} I_{P}-I_{M}\\ I_{R}-I_{L} \end{array} \end{array}\right]$$

(3) $$M=\left[\begin{array}{c} M_{00}\;\;M_{01}\;\;M_{02}\;\;M_{03}\\ M_{10}\;\;M_{11}\;\;M_{12}\;\;M_{13}\\ \begin{array}{c} M_{20}\;\;M_{21}\;\;M_{22}\;\;M_{23}\\ M_{30}\;\;M_{31}\;\;M_{32}\;\;M_{33} \end{array} \end{array}\right]\;\;$$

where Table 1 and Table 2 define each of the Stokes vector elements.

While the Stokes vector represents the polarization properties of light, the Mueller matrix contains complete and detailed information about all the polarization properties of the medium. Hence, the different polarization properties of a medium are encoded in the various elements of the Mueller matrix. When the medium under measurement exhibits a limited degree of complexity, the Mueller matrix can be interpreted from expression (Equation (4)) of the matrix where it is possible to give a physical sense in terms of the effects of the medium on the incident light [29].

(4) $$M=\left[\begin{array}{c} M_{00}=T\;\;\;M_{01}=LE\;\;M_{02}=LE^{\prime }\;M_{03}=CE\\ M_{10}=LE\;\;M_{11}=D\;\;M_{12}=CR\;\;M_{13}=LR\prime \\ \begin{array}{c} M_{20}=LE^{\prime }\;M_{21}=CR\;\;M_{22}=D^{\prime }\;\;M_{23}=LR\\ M_{30}=TE\;\;M_{31}=LR^{\prime }\;M_{32}=LR\;\;M_{33}=D \end{array} \end{array}\right]$$

where *T* is total transmitted or reflected intensity (depends on the experimental setup), *LE*, *LE*′ and *CE* are the different linear and circular extinctions respectively relating to the polarization properties, *LR*, *LR*′ and *CR* are the different linear and circular retardances, respectively, to the birefringence or retardance, and *D*, *D*′ are the different indicatives of depolarization. In this case, the three basic polarization properties considered are extinction (differential attenuation of orthogonal polarization), birefringence or retardance (de-phasing of orthogonal polarization) and depolarization on the matrix diagonal [30].

## 3. Materials and Methods

### 3.1. Liquid Optical Phantoms

#### 3.1.1. Mixture Composition

Turbid liquid samples based on a similar protocol detailed in [31] were composed of intralipid 20% solution (IL), methylene blue (MB) and distilled water used as scattering, absorbing and dilution materials, respectively. Fat emulsions such as intralipid 20% are frequently used in the research of light propagation in turbid media [32,33]. IL 20% (batch 10IB7209, Fresenius Kabi, Sèvres, France) is a sterile and non-pyrogenic fat emulsion originally prepared for intravenous feeding that contains 200 g·L^−1^ soybean oil, 12 g·L^−1^ egg phospholipids, 22 g·L^−1^ glycerin and water [34,35]. In particular, this solution contains emulsified fat particles with typical size lower than 500 nm [35,36] that act as spherical scattering particles. Considering the concentration of the different constituents, a volume concentration of 22.7% scattering particles in pure IL 20% is obtained. These fat emulsions offer some remarkable benefits over other calibration standards. Because IL is turbid and presents no strong absorption bands in the visible region of the electromagnetic spectrum, and is homogeneous, sterile, non-toxic, readily available and relatively low cost, which presents surprisingly small batch-to-batch variations that are highly stable over time and at different temperatures, IL solution is well adapted and widely used as a diffuse reference standard in many measurement systems for optical characterization [32].

MB (M9140, batch MKBR892V, Sigma-Aldrich, Saint-Quentin-Fallavier, France) is a water-soluble non-scattering dye that presents two typical absorption peaks at 609 nm and 668 nm due to dimer and monomer forms in aqueous solutions, respectively [37,38,39]. It is commonly used as an absorber for the preparation of liquid optical phantom. In contrast, the absorption by water and IL is moderate in this spectral range. As a result, MB and IL are well-adapted to discriminating the effects of scattering and absorption on the measured polarized spectra in the considered wavelength range.

#### 3.1.2. Sample Preparation

**Figure 1:**
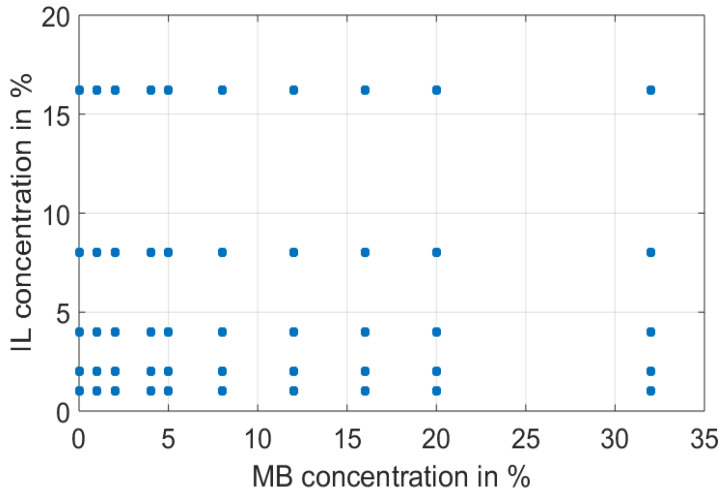
A set of 50 liquid optical phantoms obtained by mixing methylene blue (MB; absorber), intralipid 20% (IL; scatterer) and distilled water (dilution agent) prepared in this study.

A set of 50 liquid optical phantoms was prepared to cover a wide range of absorption and scattering properties. To do this, 10 concentrations of the absorber (0, 1, 2, 4, 5, 8, 12, 16, 20 and 32 mL of a 400 µM MB stock solution) with 5 concentrations of the scatterer (1, 2, 4, 8 and 16 mL of IL 20% solution) in different ratios were mixed (Figure 1). Following this, the phantoms had MB concentrations of 0, 4, 8, 16, 20, 32, 48, 64, 80 and 128 µM, and IL concentrations of 0.227, 0.454, 0.908, 1.816 and 3.682%, respectively. Eventually, distilled water was added to obtain 100 mL for all liquid optical phantoms. Finally, each sample was carefully and rigorously shaken to homogenize the aqueous solutions before making spectral measurements.

### 3.2. Polarization Spectroscopy System

#### 3.2.1. Experimental Setup

**Figure 2:**
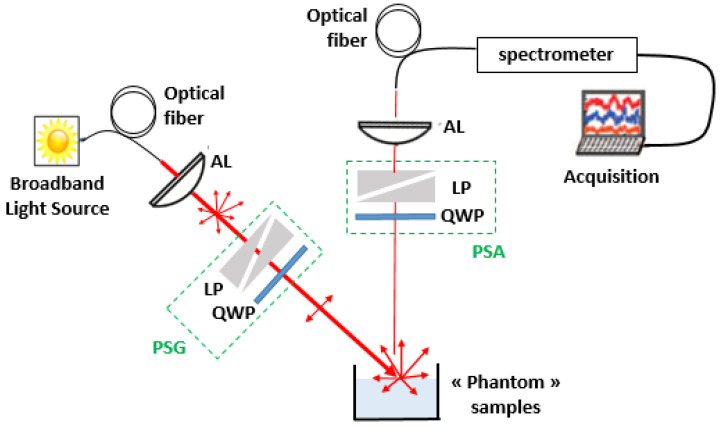
Schematic representation of PoLiS system in reflectance with PSG (polarization state generator), PSA (polarization state analyzer), LP (linear polarizer), QWP (quarter-wave plate) and AL (aspheric lens).

An optical setup was implemented to ensure the diffuse reflectance measurements in polarized light within the VIS–NIR spectral region (Figure 2). This system was based on the polarized light spectroscopy system (PoLiS) that we used in a previous publication [19]. However, for this study, the PoLiS system integrated a polarization state generator (PSG) and a polarization state analyzer (PSA) in order to generate and to select the various polarization states, which are necessary to obtain all the elements of the Mueller matrix. Both the PSG and PSA consisted of a rotating broadband linear polarizer (LP) designed in 400–700 nm spectral range (NT52-557, Edmunds Optics, Barrington, New Jersy, United States) associated with rotating quarter-wave plate (QWP) (AQWP05M-600, Thorlabs, Newton, New Jersey, United States) in the optimized spectral range 400–800 nm. A broadband halogen light source (150 W, Leica Cls) was used for illumination and raw spectra were collected with a spectrometer (MMS1, Zeiss, Oberkochen, Germany) in the 350–1100 nm wavelength range at 3 nm intervals. The illumination arm was oriented at 45° zenith angle whereas the collection arm was placed at the zenith in order to avoid specular reflection. Given the optical features of the different elements constituting the optical system, the spectral measurements were optimal in the 400–700 nm spectral range.

#### 3.2.2. Spectral Acquisition

For all phantoms, diffuse reflectance spectra in polarized light were measured for each of the 49 configurations of both PSG and PSA. Moreover, for each of them, a white diffuse standard (Spectralon^®^, SRS-99-010, Labsphere, North Sutton, United States) was applied in order to standardize spectra from non-uniformities of all components of the PoLiS system. Then, each calculated element of the matrix (M_11_, M_22_, …, M_44_) was divided by the corresponding spectral reference in order to obtain a normalized hybrid diffuse reflectance (r_11_, r_22_, …, r_44_) respectively. All data are available in this publication [27].

#### 3.2.3. Determination of Mueller Matrix Elements

**Figure 3:**
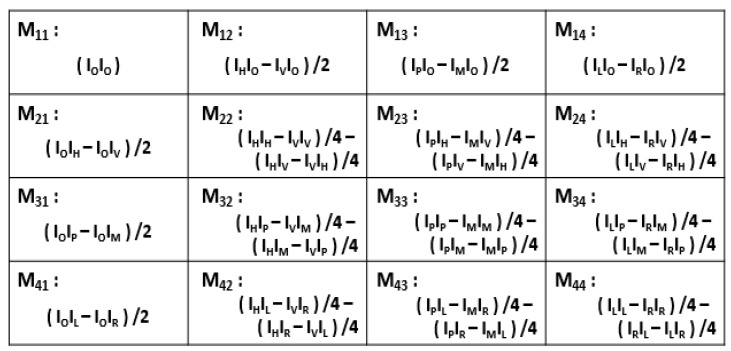
A matrix array showing the polarization measurements necessary to obtain each Mueller matrix element. A two-letter combination corresponds to one measurement. For example, the combination (I_V_I_P_) means that the PSG is adjusted to obtain linear polarization along the vertical axis (*y* axis) for incoming light and PSA is adjusted to recover linear polarization with a +45° offset for reflected light. I_0_ corresponds to unpolarized light.

The Mueller matrix was determined experimentally to provide a complete description of the response of a medium to polarized light excitation in reflection or transmission configurations. The necessary measurements and combinations listed in Figure 3 were made to determine each matrix element [25].

Thus, to determine the 16 elements of the complete Mueller matrix, it was necessary to measure 7 configurations for both the PSG and PSA, respectively, to obtain the 49 diffuse reflectance spectral measurements, i.e., 7 for incoming polarized light multiplied by 7 for reflected polarized light. From this and following Figure 3, each matrix element was calculated by the corresponding linear combination.

### 3.3. PLS Models

All multivariate data analyses were performed with Matlab software v. R2015b (The Mathworks Inc., Natick, MA, USA). A partial least square (PLS) model was used to predict the chemical and physical parameters of the liquid optical phantoms. PLS models were built from each element of the diagonal of the Mueller matrix using the whole calibration set (two-thirds of the sample) and a predicting set (one-third of the sample). The number of latent variables (LV) was determined by comparing performances using the leave-one-out cross-validation method [40]. Basic statistical parameters including the determination coefficient (R^2^), the root mean standard error of prediction (RMSE_P_) and the bias were calculated. These main parameters were used to assess the performance of each model for predicting absorber and scatterer concentrations.

## 4. Results and Discussion

### 4.1. Raw Spectra Analysis

**Figure 4:**
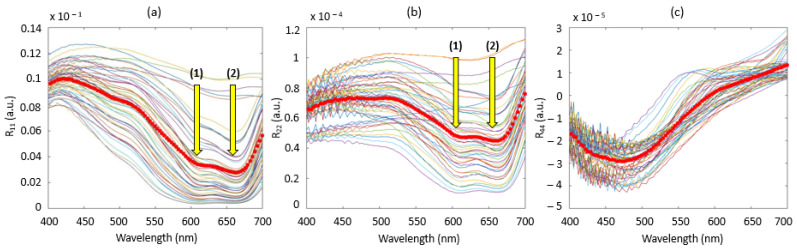
Results of r_11_ (**a**), r_22_ (**b**) and r_44_ (**c**) diffuse reflectance spectra for the M_11_, M_22_ and M_44_ elements of the Mueller matrix diagonal, respectively. The yellow arrows (1) and (2) in figures (**a**) and (**b**) indicate the two absorption peaks of methylene blue at 609 nm and 668 nm, respectively.

Figure 4 shows the results of combining the diffuse reflectance spectra in the 400–700 nm wavelength range to obtain the three specific elements of the Mueller matrix diagonal: r_11_ (Figure 4a), relative to the non-polarized light element; r_22_ (Figure 4b), specific to the linear polarization element and r_44_ (Figure 4c.), specific to the circular polarization element. In all the three figures, the thick red line represents the mean spectrum of the 50 calculated spectra in order to detect a possible trend.

**Figure 5:**
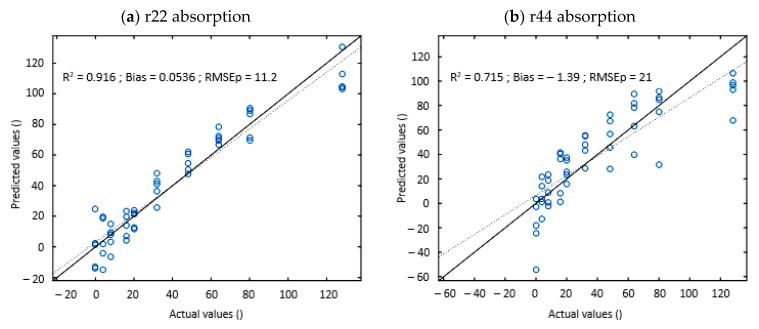

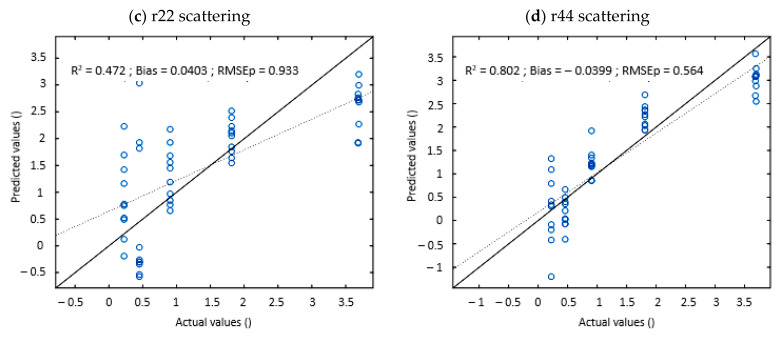
Predicted vs measured absorber concentrations (c_abs_) for (**a**) r_22_ and (**b**) r_44_, and scatterer concentrations (c_dif_) for (**c**) r_22_ and (**d**) r_44_.

The interpretation of these spectra remains delicate and complex because these spectra were obtained from different linear combinations of diffuse reflectance spectra. The spectra obtained from the r_11_ element (Figure 4a) were simply the total diffuse reflected intensity from an unpolarized incident beam. Spectra had a consistent shape in regard to the media considered: the typical spectrum of methylene blue the two absorption peaks at 668 nm and 609 nm and a translation of the baseline, which therefore indicates the mixed presence of scattering and absorption effects. For Figure 5b, relating to the r_22_ element of the Mueller matrix, we found a spectrum similar to the r_11_ element with phenomena of vertical translations of the baseline but with lower values and relatively flatter in the 400–550 nm spectral range. Moreover, information on absorption peaks at 608 and 609 nm were also preserved. All of these basic observations seem to indicate a preferential sensitivity to the absorption effect for this element. In contrast, Figure 4c, relating to the r_44_ element of the Mueller matrix, shows spectra that are closer to each other than previously. The baselines described above no longer occurred and the absorption peaks related to methylene blue in the spectral range 600–700 nm also did not appear, which could therefore suggest that only the scattering phenomenon was involved. Thus, it would seem that certain elements of the diagonal are sensitive to well-identified phenomena such as absorption or scattering effects. To confirm this assumption, PLS models from r_11_, r_22_ and r_44_ were established.

### 4.2. C_abs_ and C_dif_ Prediction Models

As the experimental system was optimized in the 400–700 nm spectral range and to respect the Rayleigh regime ($d_{max}=500\;\mathrm{nm}\leq \lambda$ with *d_max_* being the maximum diameter of the considered particles and *λ* the wavelength of the electronic radiation), models were established in the 500–700 nm restricted spectral range with focus on the diagonal elements of the Mueller matrix to determine absorber (C_abs_) and scatterer (C_dif_) concentrations. The resulting models and associated quality parameters are shown in Figure 5 and Table 2, respectively.

#### 4.2.1. Absorber Concentration Models

Quality parameters of the models to predict absorber concentration are shown in Table 3a. The best prediction quality was obtained with the r_22_ element with values of 0.92 and 11.2 for the R²_pred and RMSEp, respectively. In contrast, the r_44_ element shows the least probative results with values of 0.72 and 21 for the R²_pred and RMSEp, respectively. Moreover, as mentioned previously in the description of the obtained spectra, r_11_ and r_22_ show similar spectra with the conservation of the information related to the different absorption peaks in the 550–700 nm spectral range but with the less pronounced baseline effects in the 400–550 nm spectral range for the r_22_ element compared to r_11_. This difference of behavior in the spectra seems to improve the performance of the models with R²_predvalues of 0.92 and 0.81 for r_22_ and r_11_, respectively. Thus, these different elements tend to confirm the sensitivity of linear polarization through the r_22_ element of the Mueller matrix to the absorption properties of the medium in the Rayleigh regime. This finding has been reported many times in the literature, where the linear polarization state is most-widely used to overcome parasitic effects related to diffusion [41,42,43]. In fact, linear polarization persists through a larger number of scattering events longer than circular polarization such that circularly polarized light is more depolarized than linear polarized light in this specific regime.

#### 4.2.2. Scatterer Concentration Models

Quality parameters of the models to predict scatterer concentration are shown in Table 3b. In this case, and contrary to the previous results in Table 3a for absorption, the model based on the r_44_ element presents better predictive capacities for the C_dif_ variable with values of 0.80 and 0.564 for the R²_pred and RMSEp, respectively. In contrast, the r_22_ element clearly shows the lowest performance with values of 0.47 and 0.933 for the R²_predand RMSEp, respectively. Thus, this marked difference between r_22_ and r_44_ of the Mueller matrix suggests a greater sensitivity of the circular polarization (r_44_) compared to linear polarization (r_22_) to scattering effects induced by the physics of the medium. However, while the model for the prediction of absorber concentration from element r_22_ is probative regarding absorption properties, this is less pronounced with element r_44_ for scattering-related properties. Nevertheless, these results are to be moderated, because despite several constraints such as (i) a limited number of samples (*n* = 50) to establish robust models, (ii) no pre-processing was applied on the raw spectra to reduce undesired scatter effects such as baseline shift and non-linearity problems and (iii) a Rayleigh regime—that is not rigorously respected—with the composition of the liquid optical phantoms used in this study, the results that were achieved are very promising for the separation and characterization of the media according to their scattering and absorption properties.

Thereafter, as the r_22_ and r_44_ elements of the Mueller matrix appear to be the most relevant for predicting absorber and scatterer concentration, respectively, these two elements were focused on. A comparison of the prediction models for the absorber concentrations (C_abs_) established with r_22_ and r_44_ is represented by Figure 5a,b, respectively. For the r_22_ model, the results obtained are significant with an R^2^ above 0.90 and a moderate bias. However, we can observe a minor overestimation for concentrations between 30 µM and 70 µM. For the r_44_ model, we can observe pronounced dispersion, which is translated by a lower R^2^ around 0.70 and an accentuated bias caused by an underestimation of the extreme concentration values.

At last, a comparison of the prediction models for the scatterer concentrations (C_dif_) established with r_22_ and r_44_ is represented by Figure 5c,d, respectively. It appears that for both models the results are not satisfying, especially at low concentrations (<1%) with high dispersion. In addition, both models show a trend of overestimating or underestimating the measured value from concentrations above 1%. It should also be noted that although the r_44_ element provides the best model, it seems to be less efficient than r_22_ for low-concentration values between 0 and 0.5% because we can see a more pronounced dispersion of predicted values. For this reason, although circular polarization through r_44_ seems to be more sensitive to scattering phenomena, its use should be carefully considered depending on the application and the concentrations involved.

## 5. Conclusions

In this study, the objective was to assess absorption and scattering properties in complex media by using an original approach that mixes polarized light spectroscopy and the Mueller matrix concept coupled with PLS models. The well-known properties of liquid optical phantoms were used to test this combined approach. The results obtained show that the proposed approach is consistent with separate absorption and scattering effects. In fact, model results based on the diagonal elements have helped to highlight the sensitivity of the r_22_ and r_44_ matrix elements to absorption and scattering components, respectively, through the good prediction of chemical and physical parameters, especially in the Rayleigh regime. It would be interesting to extend this study to each of the other 12 elements that could be tested and evaluated to obtain further information about the absorbing and scattering properties of turbid media: refractive index, particle shape and size, etc. In addition, it would also be interesting to explore two ways of improving the prediction accuracy and robustness of the models: (i) testing different scatter-correction methods such as MSC (multiplicative scatter correction), SNV (standard normal variate) and normalization that are pre-processed on spectra dedicated to reduce physical variability and to adjust baseline shift between samples present on the majority of our diffuse reflectance spectra and (ii) combining multiple elements of the Mueller matrix in prediction models by using different PLS methods such as multi-block regression (SO-PLS, PO-PLS, etc.) [44,45]. This approach could be implemented and tested in different sensing applications as an analysis technique for quality control (chemical composition, detection of traces or residues, etc.) of a wide diversity of media and products for biomedical or food production domains, for example.

## Figures and Tables

**Table 1:** Meaning of the four Stokes parameters.

Abbreviation	Meaning
I	Total light intensity corresponding to addition of any two orthogonal components
Q	Portion of the intensity corresponding to the difference between vertical and horizontal polarization state
U	Portion of the intensity corresponding to the difference between intensities of linear +45° and −45°
V	Portion of the intensity corresponding to the difference between right circular and left circular polarization states

**Table 2:** Meaning of the different abbreviations related to the polarization state following six intensity measurements.

Abbreviation	Meaning
I_H_	Linearly polarized along the horizontal axis (0°)
I_V_	Linearly polarized along the vertical axis (90°)
I_P_	Linearly polarized with a +45° offset
I_M_	Linearly polarized with a −45° offset
I_R_	Right-handed circularly polarized
I_L_	Left-handed circularly polarized

**Table 3:** Model performances built from the diagonal elements of the Mueller matrix for (a) C_abs_ (absorber concentrations) and (b) C_dif_ (scatterer concentrations).

(a)				
		C_abs_		
	LV	R^2^_pred	Bias_pred	RMSEp
r_11_	5	0.81	−1.64	16.8
r_22_	5	0.92	0.05	11.2
r_33_	6	0.75	−2.47	19.9
r_44_	6	0.72	−1.33	21
(b)				
		C_dif_		
	LV	R^2^_pred	Bias_pred	RMSEp
r_11_	5	0.75	0.014	0.647
r_22_	6	0.47	0.040	0.933
r_33_	6	0.61	−0.083	0.871
r_44_	5	0.80	0.040	0.564

## Data Availability

The data presented in this study are openly available in Journal Data in Brief at https://doi.org/10.1016/j.dib.2019.103756 (accessed on 5 November 2022).
